# Genetic evidence implicating natriuretic peptide receptor-3 in cardiovascular disease risk: a Mendelian randomization study

**DOI:** 10.1186/s12916-023-02867-x

**Published:** 2023-04-26

**Authors:** Héléne T. Cronjé, Ville Karhunen, G. Kees Hovingh, Ken Coppieters, Jens O. Lagerstedt, Michael Nyberg, Dipender Gill

**Affiliations:** 1 Department of Public Health, Section of Epidemiology, University of Copenhagen, Copenhagen, Denmark; 2 Faculty of Science, Research Unit of Mathematical Sciences, University of Oulu, Oulu, Finland; 3 Research Unit of Population Health, Faculty of Medicine, University of Oulu, Oulu, Finland; 4 Department of Vascular Medicine, Amsterdam University Medical Centers, University of Amsterdam, Amsterdam, The Netherlands; 5 Global Chief Medical Office, Novo Nordisk, Copenhagen, Denmark; 6 Global Project Management, Global Drug Discovery, Novo Nordisk, Copenhagen, Denmark; 7 Rare Endocrine Disorders, Research and Early Development, Novo Nordisk, Copenhagen, Denmark; 8 Department of Experimental Medical Science, Lund University, 221 84 Lund, Sweden; 9 Vascular Biology, Research and Early Development, Novo Nordisk, Maaloev, Denmark; 10 Chief Scientific Advisor Office, Research and Early Development, Novo Nordisk, Copenhagen, Denmark; 11 Department of Epidemiology and Biostatistics, School of Public Health, Imperial College London, London, UK

**Keywords:** Blood pressure, C-type natriuretic peptide, Cardiovascular disease, Genetic epidemiology, Mendelian randomization, NPR2, NPR3

## Abstract

**Background:**

C-type natriuretic peptide (CNP) is a known target for promoting growth and has been implicated as a therapeutic opportunity for the prevention and treatment of cardiovascular disease (CVD). This study aimed to explore the effect of CNP on CVD risk using the Mendelian randomization (MR) framework.

**Methods:**

Instrumental variables mimicking the effects of pharmacological intervention on CNP were identified as uncorrelated genetic variants located in the genes coding for its primary receptors, natriuretic peptide receptors-2 and 3 (NPR2 and NPR3), that associated with height. We performed MR and colocalization analyses to investigate the effects of NPR2 signalling and NPR3 function on CVD outcomes and risk factors. MR estimates were compared to those obtained when considering height variants from throughout the genome.

**Results:**

Genetically-proxied reduced NPR3 function was associated with a lower risk of CVD, with odds ratio (OR) 0.74 per standard deviation (SD) higher NPR3-predicted height, and 95% confidence interval (95% CI) 0.64–0.86. This effect was greater in magnitude than observed when considering height variants from throughout the genome. For CVD subtypes, similar MR associations for NPR3-predicted height were observed when considering the outcomes of coronary artery disease (0.75, 95% CI 0.60–0.92), stroke (0.69, 95% CI 0.50–0.95) and heart failure (0.77, 95% CI 0.58–1.02). Consideration of CVD risk factors identified systolic blood pressure (SBP) as a potential mediator of the NPR3-related CVD risk lowering. For stroke, we found that the MR estimate for NPR3 was greater in magnitude than could be explained by a genetically predicted SBP effect alone. Colocalization results largely supported the MR findings, with no evidence of results being driven by effects due to variants in linkage disequilibrium. There was no MR evidence supporting effects of NPR2 on CVD risk, although this null finding could be attributable to fewer genetic variants being identified to instrument this target.

**Conclusions:**

This genetic analysis supports the cardioprotective effects of pharmacologically inhibiting NPR3 receptor function, which is only partly mediated by an effect on blood pressure. There was unlikely sufficient statistical power to investigate the cardioprotective effects of NPR2 signalling.

**Supplementary Information:**

The online version contains supplementary material available at 10.1186/s12916-023-02867-x.

## Background

Cardiovascular disease (CVD) is a leading cause of morbidity and mortality globally, with approximately 523 million prevalent cases and 19 million deaths in 2019 [1]. The burden is steadily rising, and the number of years lived with CVD-related disability worldwide almost doubled between 1990 and 2019, from 18 to 34 million. There is therefore a continued imperative to identify novel therapeutic targets for the prevention and treatment of CVD.

Human genetic data offer the opportunity to efficiently investigate the potential of proteins as therapeutic targets for preventing and treating disease [2]. This is based on the central dogma that genes code for proteins. It follows that natural variation in the genes coding for these proteins can be used to predict the effect of pharmacologically perturbing the corresponding protein target. As genetic variants are randomly allocated at conception, their association with clinical outcomes are relatively devoid of the environmental confounding and reverse causation bias that can hinder causal inference in traditional epidemiological studies. This Mendelian randomization (MR) paradigm can be used to support causal inferences related to the effects of therapeutic intervention [3]. The use of human genetic data additionally offers advantages over animal models in terms of organism relevance, and further, the current availability of large-scale human genetic association data for a range of clinical traits means that such analyses can be undertaken relatively quickly and cost-effectively.

C-type natriuretic peptide (CNP) belongs to the family of natriuretic peptides including atrial natriuretic peptide (ANP) and B-type natriuretic peptide (BNP), which are collectively involved in regulating the structure and function of the cardiovascular system [4–6]. In accordance with this role of CNP in the cardiovascular system, preclinical observations have provided evidence for perturbations in CNP signalling being involved in the development of CVD [7–9]. Specifically, CNP is postulated to help regulate cardiac structure and function, microcirculatory blood flow and systemic blood pressure [7–9]. CNP induces its effects through two receptors — natriuretic peptide receptor (NPR) 2 and 3. NPR2 is a particulate guanylyl cyclase that catalyses the synthesis of cyclic guanosine monophosphate upon binding by CNP to increase protein kinase G signalling. NPR3 clears all three natriuretic peptides via a receptor-mediated internalization and degradation process [10] but has also been reported to have a signalling function [6]. Mutations in both NPR2 [11] and NPR3 [12] have been associated with altered height. The latter observation is likely to primarily reflect a loss of NPR3 function leading to decreased clearance of CNP and a consequent increase in NPR2-mediated bone growth [13, 14]. Accordingly, CNP analogues have demonstrated efficacy for increasing growth in individuals with achondroplasia [15]. As such, it follows that the phenotype of height may be used as a clinically validated trait by which to identify genetic variants in the *NPR2* and *NPR3* genes that mimic the effects of pharmacologically targeting NPR2 and NPR3 signalling and function.

In this study, we leverage genetic variants mimicking NPR2 signalling and NPR3 and function as instrumental variables for studying the effects of CNP on CVD subtypes and risk factors within the MR paradigm. By following up any MR associations with statistical colocalization analyses, we demonstrate the robustness of our findings against confounding by variants in linkage disequilibrium (LD). Our findings are of direct translational relevance to clinical development efforts of CNP analogues for the prevention and treatment of CVD.

## Methods

### Study overview

**Fig. 1:**
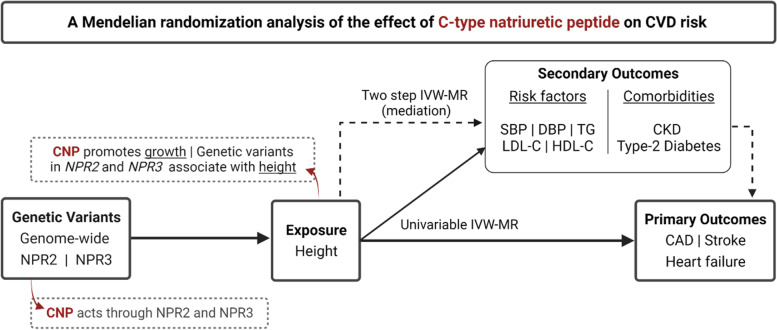
Study design overview (created with BioRender.com). CAD, coronary artery disease; CKD, chronic kidney disease; CNP, C-type natriuretic peptide; DBP, diastolic blood pressure; HDL-C, high-density lipoprotein cholesterol; IVW-MR, inverse variance weighted mendelian randomization; LDL-C, low-density lipoprotein cholesterol; NPR2, natriuretic peptide receptor 2; NPR3, natriuretic peptide receptor 3; SBP, systolic blood pressure; T2DM, Type-2 diabetes mellitus; TG, triglycerides

Given the known effect of CNP on NPR2 signalling and NPR3 function [7, 8], and that increased NPR2 signalling and reduced NPR3 function are associated with increased height, we leveraged uncorrelated genetic variants related to height at these gene regions as genetic instruments for pharmacological perturbation of those targets. These genetic variants were then used within the MR paradigm for studying the effects of NPR2 signalling and NPR3 function on the risk of CVD, comprising coronary artery disease (CAD), stroke, and heart failure (HF). In follow-up analyses, we investigate their associations with related cardiometabolic risk factors and comorbidities to interrogate underlying mechanisms. MR estimates for the *NPR2* and *NPR3* gene regions were compared to those obtained for height variants from throughout the genome. Secondary analyses were performed to investigate whether mediating cardiometabolic risk factors, namely blood pressure, might explain any MR evidence supporting effects of NPR2 or NPR3 on CVD outcomes. For outcomes where there was MR evidence supporting causal effects, colocalization analysis was performed to investigate whether the findings may be attributable to confounding through a variant in LD [16]. The study overview is schematically depicted in Fig. 1.

### Instrument selection

Genetic instruments for height were selected from a multi-ancestry meta-analysis of 281 genome-wide association studies (GWASs), representing more than 5 million individuals (Table 1, [17, 18]). To identify genetic variants affecting height by any mechanism, all single-nucleotide polymorphisms (SNPs) that associated with height at *p* < 5 × 10^−08^ were retrieved and pruned to pairwise R^2^ = 0.1 using the 1000G European reference panel. We generated instruments for the *NPR2* and *NPR3* gene loci by restricting height-associated variants to the relevant gene loci ± 100 kb; chr9:35,792,151–35,809,729 for *NPR2* and chr5:32,689,176–32,791,819 for *NPR3* (Ensembl, build GRCh37/hg19), followed by pruning to pairwise *R*^2^ = 0.1, again using the 1000G European reference panel.

**Table 1:** Included genome-wide association data sources

Trait	Trait definition and ascertainment	Unit	Ancestral inclusion	Total *N* (cases *N*)	Study and data source
Height	Measured or self-reported	Per SD (cm)	76% EUR, 9% EAS, 8% HIS, 5% AFR, 1% SAS	5,380,080	[17, 18]
ANP	Relative aptamer abundance in plasma	Per SD (RFU)	100% EUR	35,559	[19, 20]
BNP					[19, 21]
NT-pro-BNP	Plasma Olink proximity extension assay	Per SD (NPX)	100% EUR	21,758	[22, 23]
CAD	ACS, CAD, or MI. Medical records^a^ or self-report	Case status^b^	~ 85% EUR, 15% EAS	1,378,170 (210,842)	[24, 25]
Stroke	All-cause. Medical records or self-report	Case status^b^	67% EUR, 25% EAS, 4% AFR, 3% SAS, 1% HIS	1,614,080 (110,182)	[25, 26]
HF	Medical records. Study-based adjudicated self-report	Case status^b^	> 99% EUR	977,323 (47,309)	[27, 28]
T2DM	Study-based diagnosis, medical records, or self-report	Case status^b^	51% EUR, 28% EAS, 8% SAS, 7% AFR, 6% HIS	1,339,889 (180,834)	[29, 30]
CKD	Study-based, eGFR < 60 mL/min/1.73m^2^	Case status^b^	~ 77% EUR	625,219 (64,164)	[31, 32]
LDL-C	Fasting or non-fasting. Adjusted for medication: LDL-C ÷ 0.7, TG ÷ 0.8	Per SD (mg/dL)	80% EUR, 9% EAS, 6% AFR, 3% HIS, 2% SAS	1,654,960	[33, 34]
HDL-C					[33, 35]
TG					[33, 36]
SBP	Mean of two seated measures taken a few moments apart	Per SD (mmHg)	100% EUR	317,756	[37–39]
DBP				317,756	[37, 38, 40]

*AFR* Admixed African/African, *ACS* acute coronary syndrome, *ANP* atrial natriuretic peptide, *BNP* brain/B-type natriuretic peptide, *CAD* coronary artery disease, *CKD* chronic kidney disease, *DBP* diastolic blood pressure, *eGFR* estimated glomerular filtration rate, *EUR* European, *EAS* East Asian, *HDL-C* high-density lipoprotein cholesterol, *HIS* Hispanic, *LDL-C* low-density lipoprotein cholesterol, *MI* myocardial infarction, *NPX* Normalized Protein eXpression, *NT-proBNP* N-Terminal Pro-B-Type Natriuretic Peptide, *RFU* relative fluorescence units, *SAS* South Asian, *SBP* systolic blood pressure, *SD* standard deviation, *T2DM* type-2 diabetes mellitus, *TG* triglycerides. ^a^Medical records include inpatient and outpatient diagnostic registers, medication prescriptions, surgical procedures, death certificates, or medical insurance registries. ^b^Includes prevalent and incident cases

### Outcomes

Genetic association estimates of CAD, stroke, HF, chronic kidney disease (CKD), type-2 diabetes mellitus (T2DM), and blood lipids [low-density lipoprotein cholesterol (LDL-C), high-density lipoprotein cholesterol (HDL-C), and triglycerides (TG)] were obtained from the largest available published GWASs that made their data publicly accessible [24–36]. For systolic and diastolic blood pressure (SBP and DBP) we relied on data from the UK Biobank [37–40] to circumvent collider bias introduced by the adjustment for body mass index (partly defined by our exposure of interest: height) in the largest publicly available GWAS on blood pressure traits [41]. While all data sources overrepresented individuals of European ancestry, only three were limited to European data specifically (Table 1).

### Statistical analysis

Random-effects inverse-variance weighted MR was performed to explore the effect of NPR2 signalling and NPR3 function on the selected outcomes and compare these estimates to those obtained when considering height variants selected from throughout the genome. Individual MR estimates for each variant were generated with the Wald ratio, which is the ratio of the variant-outcome associations over the variant-exposure associations. Standard errors for individual variant MR estimates were estimated as the standard error of the variant-outcome association divided by the variant-exposure association.

Random-effects inverse-variance weighted meta-analysis was used to pool MR estimates across the variants comprising each exposure (i.e., height generally, NPR2-predicted height, and NPR3-predicted height), and across the primary (i.e., CAD, stroke, HF, and a composite risk estimate of these three for overall CVD) and secondary (i.e., cardiometabolic risk factors and comorbidities) outcomes. Follow-up analyses were performed for instances where either NPR2 or NPR3 demonstrated MR evidence of protective effects on CVD subtypes in the main analysis, to delineate potential underlying mechanisms. Specifically, we considered the same criteria for selecting NPR2 or NPR3 instruments as in the main analysis, but instead of height, considered SBP as the biomarker using data from the same UK Biobank study described above [37]. These MR estimates were then compared to those obtained when considering SBP variants from throughout the genome: all 379 SNPs that associated with SBP at *p* < 5 × 10^−08^ after pruning to pairwise *R*^2^ = 0.1 using the 1000G European reference panel, collectively explaining approximately 4.6% of the variance in SBP.

Similarly, we investigated whether causally relevant instruments were associated with relative circulating ANP, BNP and N-terminal pro-BNP, and whether these peptides potentially contributed to the observed instrument-CVD effects.

To investigate whether the MR associations were driven by confounding by LD, we conducted colocalization analysis for the MR findings at *p* < 0.05. Bayesian colocalization method ‘coloc’ was used to examine the genetic associations between the exposure-outcome pairs in the corresponding genetic locus [42]. While using ‘Coloc’, we assume that at most one causal variant for a trait lies within the locus, and under this assumption, the method enumerates across all the possibilities to test the competing hypotheses of H0: no causal variants; H1: causal variant for exposure only; H2: causal variant for outcome only; H3: distinct causal variants for exposure and outcome; and H4: shared causal variant for exposure and outcome. A high posterior probability (PP) for H4 (PPH4) would suggest colocalization which supports the MR results, while a high PP for H3 (PPH3) would suggest that the observed MR results may be due to LD between separate causal variants for the traits. We used the default priors of 10^−4^, 10^−4^ and 10^−5^ for a variant being associated with exposure, outcome, and both traits, respectively [16]. PPH4 > 0.5 was used as the threshold for evidence of colocalization, signifying that colocalization is more likely than any other scenario combined. To investigate the presence of pleiotropy in our primary MR findings, we used I^2^ heterogeneity estimates, and report weighted median and MR-Egger estimates MR estimates that are more robust to pleiotropic effects [43].

The discrepancies in the MR estimates using variants within the *NPR2* gene, within the *NPR3* gene, and from throughout the genome might be attributable to variation in the number of variants available to instrument each of these exposures. To examine this possibility, we iteratively sampled (without replacement) the minimum number of variants available for any of the three exposures from the full pool of variants available for the remaining two exposures respectively, and repeated random-effects inverse-variance weighted MR analysis 100 times. The mean estimate and 95% confidence interval (CI) of the 100 iterations are used to infer approximately what the MR estimates would have been if an equal number of variants were available for all exposures.

### Reporting

For MR of binary outcomes, we report odds ratios (ORs) and 95% CI per standard deviation (SD) increase in height, with 1-SD corresponding to 9.2 cm. MR estimates on continuous outcomes are reported as SD change per 1-SD height. Every 1-SD change in SBP corresponds to 19.4 mmHg, and DBP to 10.5 mmHg. For the primary MR analysis of CVD risk, we considered the association to be statistically significant if at a *p* < 0.05. For follow-up exploratory analyses exploring CVD subtypes and risk factors, we also used a threshold of *p* < 0.05 to support evidence of association. All analyses were performed on R version 4.2.1. Pruning of genetic variants was performed using the *clump_data* command of the TwoSampleMR package [44].

## Results

Our genetic instrument for height included 9695 SNPs throughout the genome, with 4 and 12 variants used to comprise instruments for NPR2 and NPR3, respectively (Additional file 1: Table S1). Collectively, the SNPs included in the genome-wide instruments explained 48.8% of the variance in height. Our NPR2 and NPR3 instruments contributed a respective 0.045% and 0.32%. All F statistics were larger than 10, and well over the threshold used for identifying weak MR instruments (Additional file 1: Table S1).

**Fig. 2:**
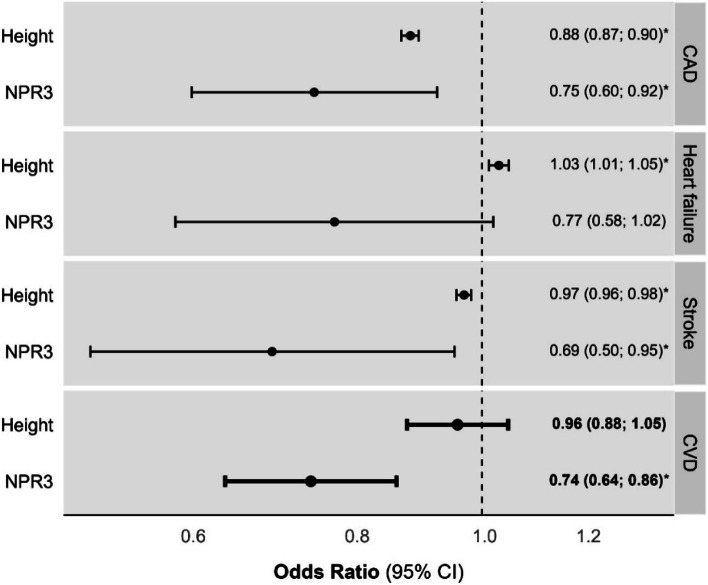
Mendelian randomization estimates of genome-wide and NPR3-predicted height on cardiovascular disease risk overall and across subtypes. Mendelian randomization estimates are scaled per standard deviation (9.2 cm) greater height. An asterisk denotes *p* < 0.05. CAD, coronary artery disease; CI, confidence interval; CVD, cardiovascular disease (reflects a pooled CAD, heart failure and stroke effect estimate); NPR3, natriuretic peptide receptor 3

The complete set of MR results are presented in Additional file 1 (Table S2) and Additional file 2 (Fig. S1 and Fig. S2). NPR2-predicted height did not robustly associate with the risk of any CVD outcomes. For NPR3-predicted height, every 1-SD increase was associated with a 26% reduction in CVD risk (Fig. 2, OR: 0.74, 95% CI: 0.64–0.86, *P* = 1.0E^−04^). When investigating CVD subtypes, NPR3- and genome-wide predicted height negatively associated with CAD and stroke risk. Although the estimate for HF was similar in magnitude, it did not reach nominal statistical significance (Fig. 2).

**Fig. 3:**
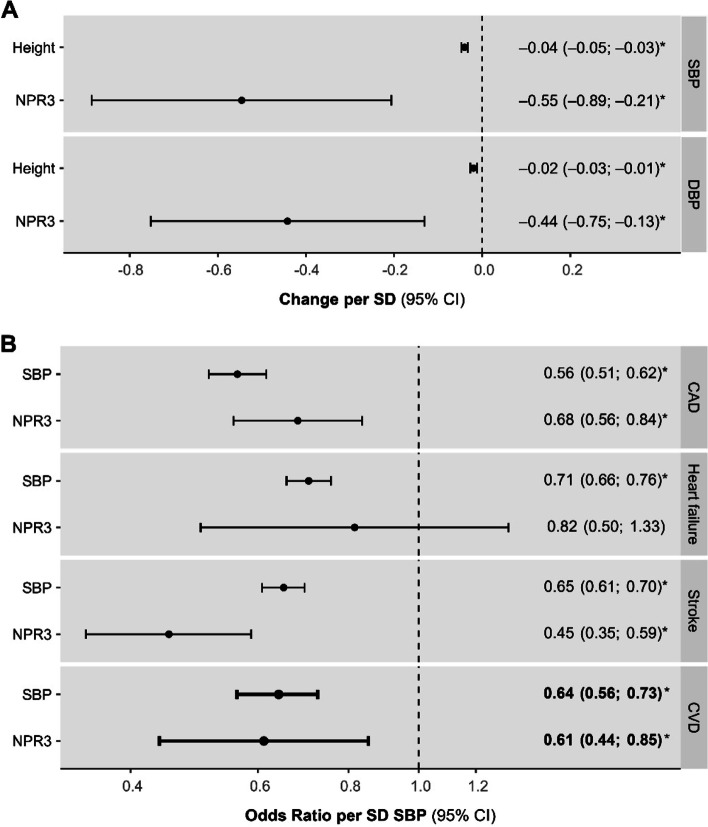
Mendelian randomization analysis considering effects through blood pressure.** A** The association of genome-wide and NPR3-predicted height with SBP and DBP. **B** The association of genome-wide and NPR3-predicted SBP reduction on CVD risk. Effect estimates are interpreted as standard deviation (SD) difference per 1-SD greater height (~ 9.2 cm) for **A**, and difference in disease odds per 1-SD lower SBP (19.4 mmHg) for **B**. An asterisk denotes *p* < .05. CAD, coronary artery disease; CI, confidence interval; CVD, cardiovascular disease (reflects a pooled CAD, heart failure and stroke effect estimate); NPR3, natriuretic peptide receptor 3; SBP, systolic blood pressure

The NPR3 and genome-wide instruments both associated with lower SBP and DBP, with NPR3 having associations that were greater in magnitude (Fig. 3A). Every 1-SD higher NPR3-predicted height corresponded to 10.6 mmHg (95% CI, 4.0–17.1 mmHg) lower SBP and 4.66 mmHg (95% CI, 1.38–7.92 mmHg) lower DBP (*P* = 2.0E^−03^ and 5.0E^−02^). To evaluate whether the effect of NPR3 signalling on CVD was mediated by blood pressure effects, we compared the causal effect of genome-wide and NPR3-restricted instrumented SBP on CVD outcomes (Additional file 1: Table S3 and Table S4). We found that genome-wide predicted SBP effect estimates overlapped with those of NPR3 signalling on CAD, HF and composite CVD. For stroke, however, the effect of NPR3-predicted SBP was greater than the effect of genome-wide predicted SBP; i.e., the NPR3-stroke effect was larger than what could be explained by a mediatory SBP effect alone (Fig. 3B, Additional file 1: Table S4).

For the remaining cardiometabolic traits (Additional file 1: Table S2 and Additional file 2: Fig. S1 and Fig. S2), genetically predicted height variants from throughout the genome associated with lower T2DM risk, higher CKD risk, and lower LDL-C and TG levels. The variants for NPR2-predicted height associated with lower HDL-C and higher TG levels. Finally, variants for NPR3-predicted height associated with lower TG. We did not observe evidence of an effect of NPR3 signalling on relative circulating ANP or BNP aptamer levels (MR estimates per 1-SD higher NPR3-predicted height: 0.17 and 0.05, 95% CIs: − 0.24 to 0.58 and − 0.24 to 0.33, *P* = 0.41 and 0.76 for ANP and BNP, respectively). Similarly, we did not find evidence of an effect of NPR3 on circulating N-terminal pro-BNP levels (MR estimates per 1-SD higher NPR3-predicted height: − 0.40, 95% CI − 1.06 to 0.26, *P* = 0.23).

**Fig. 4:**
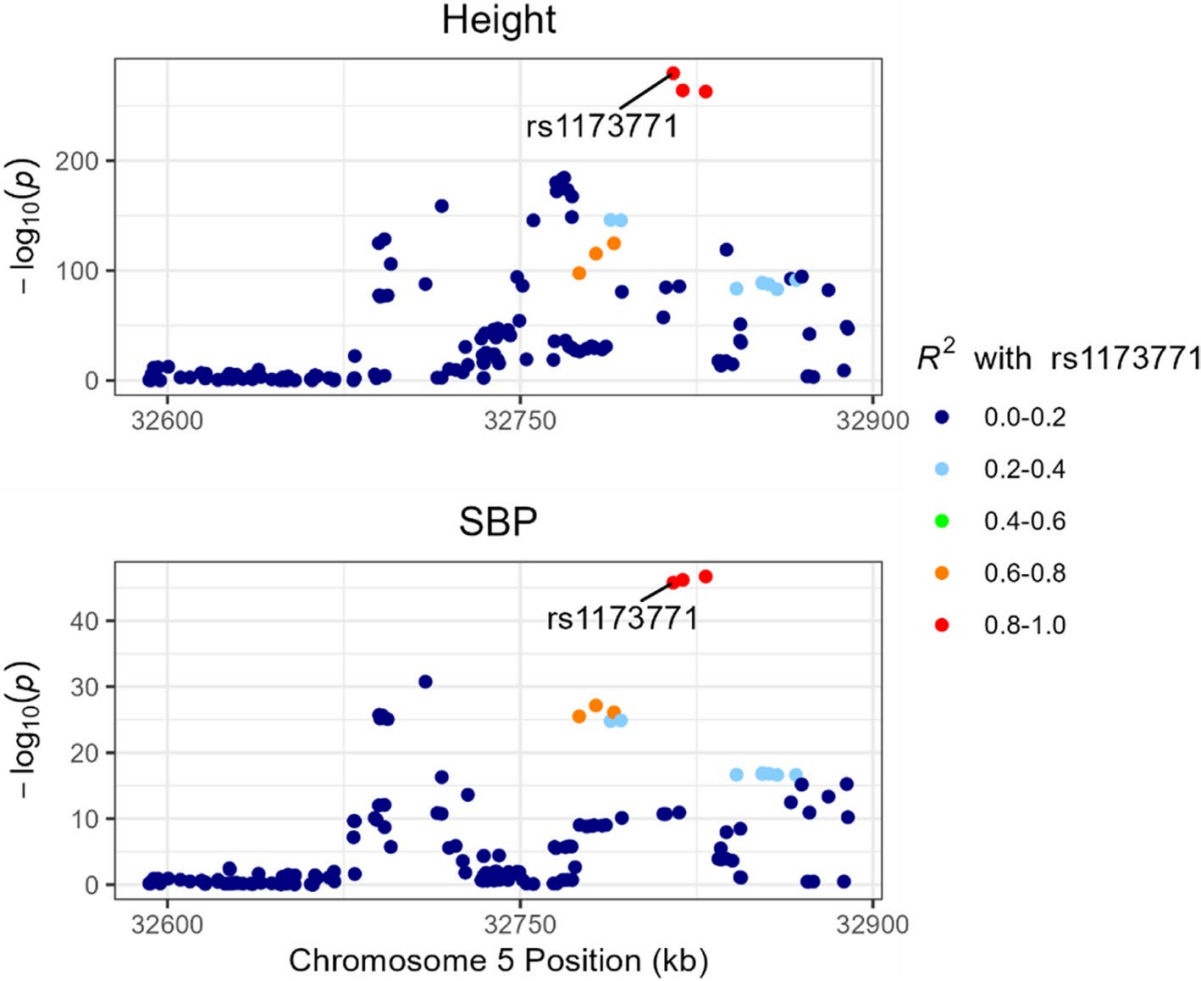
Regional plot of the genetic associations of height (upper panel) and systolic blood pressure (SBP; lower panel) within + / − 100 kb of the *NPR3* gene. The *x*-axis shows genomic position (build hg19) for each variant, and *y*-axis shows the –log10(*p*-value) for the association. Colour denotes the linkage disequilibrium *R*^2^ with rs1173771, the most likely shared causal variant based on colocalization results

Colocalization results at *NPR2* (Additional file 1: Table S5) provided evidence for a shared causal variant between height and HDL-C (PPH4 = 0.86), and height and TG (PPH4 = 0.75) (Additional file 2: Fig. S3 and Fig. S4). At *NPR3*, a causal variant was shared between height and SBP (PPH4 = 0.80, Fig. 4), height and DBP (PPH4 = 0.97), and height and stroke (PPH4 = 0.99). For height and CAD at *NPR3*, there was weaker evidence of a shared causal variant (PPH4 = 0.49) than there was for an association with only height (PPH1 = 0.50) (Additional file 2: Fig. S5, Fig. S6, and Fig. S7). There was no evidence of colocalization or distinct causal variants for height and TG at *NPR3* (PPH4 and PPH3 < 0.01, Additional file 2: Fig. S8).

Randomly sampling 4 variants from the full set of 12 for NPR3 and 9695 for height, and iteratively repeating MR analyses 100 times gave mean MR estimates of 9.1 mmHg (95% CI − 5.5–23.7 mmHg) lower SBP per 1-SD increase in NPR3-predicted height, and 1.3 mmHg (95% CI − 8.8–11.4 mmHg) lower SBP per 1-SD increase in genome-wide predicted height (with the full distribution of estimates shown in Additional file 2: Fig. S9 and Fig. S10)*.* These compare with the main MR result of 1.2 mmHg (95% CI − 5.1–7.4 mmHg) higher SBP per 1-SD increase NPR2-predicted height. When restricting to only 4 variants, estimates for all three exposures overlapped with the null, suggesting that such few variants do not offer sufficient statistical power for these analyses.

Finally, we did not observe meaningful differences between our main and sensitivity analysis-based MR estimates for the effect of NPR3 on CAD, HF, stroke, and SBP. Although there was heterogeneity observed across MR estimates generated when considering different NPR3 variants related to height for the outcomes of CAD, stroke and SBP (Additional file 1: Table S6), this did not appear to translate to biased MR estimates. Lack of bias related to the presence of pleiotropic variants was further supported by non-statistically significant corresponding NPR3-outcome MR-Egger intercepts (Additional file 1: Table S6, Additional file 2: Fig. S11, Fig. S12, Fig. S13, and Fig. S14).

## Discussion

In this work, we leverage the known effects of NPR2 and NPR3 on height to identify genetic variants to serve as instrumental variables for studying the physiological effects of CNP. Our primary MR analyses support the effects of a reduced NPR3-mediated clearance function on reducing CVD risk, with analyses across CAD, stroke and heart failure producing similar MR estimates. Exploring cardiometabolic risk factors that may explain these effects, we find strong MR and colocalization evidence implicating NPR3 in the reduction of stroke risk, related to blood pressure lowering. We did not find evidence of bias related to a departure from the no pleiotropy assumption of MR in the effect of the NPR3 on cardiovascular disease risk or SBP. Subsequent weighting of the MR analysis for SBP lowering effects suggested that this only partly explained the effects of NPR3 on reducing stroke risk, supporting that other NPR3-related mechanisms may also be at play.

Our findings are consistent with existing clinical and preclinical evidence supporting a protective role of CNP signalling on cardiovascular structure and function [5, 6]. Our consideration of height as the trait by which to identify genetic variants as instrumental variables for the effects of NPR2 and NPR3 is consistent with their known clinical effects [7, 8], and adds biological plausibility to their validity. We further compared MR estimates for variants at the *NPR2* and *NPR3* genes with those obtained when considering height variants from throughout the genome, to explore to what degree our findings are related to height itself compared to alternative target-specific mechanisms. For the traits of SBP and DBP, we found that the MR estimates for NPR3 were greater than those obtained when considering height variants from throughout the genome, suggesting that the findings cannot be explained by height effects alone. Similarly, when considering the outcome of stroke, the MR estimates for the effect of NPR3 weighted by its effect on SBP (NPR3-instrumented SBP) were greater in magnitude than those for the effect of SBP alone (genome-wide instrumented SBP), suggesting the involvement of additional NPR3-specific mechanisms.

Additional to a role of CNP, the effects of NPR3 could also be mediated via changes in endogenous levels of ANP and BNP, as perturbations in this part of the natriuretic peptide systems are also important in the aetiology of CVD [4, 45, 46]. An increase in the levels of the three peptides because of reduced NPR3-mediated clearance could potentially reduce CVD risk in a blood pressure-independent manner via anti-hypertrophic, anti-apoptotic, anti-fibrotic and/or anti-inflammatory effects directly related to natriuretic peptide signalling in the cardiovascular system [47]. However, this mechanism was not supported by our analyses investigating the effects of NPR3 signalling on relative circulating ANP or BNP levels. While it is plausible that these proteins are not altered in response to higher or lower NPR3 signalling, we also note the limited sensitivity of aptamer-based ANP and BNP quantification [48–50], and encourage further exploration of their involvement in sufficiently powered cohorts with peptide levels quantified using gold-standard methods.

Irrespective of the mechanisms underlying the results, our findings highlight the relevance of pharmacologically targeting NPR3 for prevention and treatment of CVD. Of note, increasing CNP levels and signalling through NPR3 targeting is not expected to affect bone structure and function as CNP induces linear bone growth through its effect on endochondral ossification in the growth plates that typically close near the end of puberty [13].

We found that the absence of NPR2-predicted height associations with risk of CVD may be explained by fewer genetics instruments being available for this exposure — only 4 variants, as compared to 12 for NPR3 and 9695 for height when considering the whole genome. Iteratively sampling 4 variants from the pool of NPR3 and whole-genome height variants and performing MR analyses 100 times also gave mean estimates that had 95% CIs overlapping with the null. Although the MR associations of NPR2-predicted height with HDL-C and TG were corroborated in colocalization, their clinical relevance is unclear.

This work has several strengths. By using the MR paradigm, our findings are less vulnerable to the environmental confounding and reverse causation bias that can hinder causal inference in traditional epidemiological study designs. Additionally, by incorporating genetic association summary data we use human-centric analysis with more directly translatable findings, thereby bypassing the key limitation of animal models [2]. Finally, the use of publicly available genetic association summary data makes our approach maximally time and cost-efficient.

Our work also has limitations. Despite the corroborating colocalization and sensitivity analyses supporting that the MR findings are unlikely to be attributable to genetic confounding through a variant in LD [16], there remains the possibility that the MR estimates may be biased by the pleiotropic effects of the genetic variants employed as instruments. Unfortunately, this possibility can never be entirely excluded.

We also acknowledge that while the colocalization method employed here has been shown to be generally robust to the presence of multiple causal variants, it assumes only a single causal variant, in contrast to our MR analysis. We were also motivated to use Coloc despite this assumption as it enabled us to rely on published summary data without an LD matrix representative of the exposure and outcome GWAS estimates, which other methods would not [16].

Additionally, our approach only captures the potential effects of NPR2 and NPR3 that share the same signalling mechanism as those that affect height. If these proteins are affecting CVD risk through separate mechanisms, our instruments may not be sensitive to this [51]. As discussed above, our approach also likely had insufficient statistical power for study of NPR2-related mechanisms. Finally, our analyses were largely confined to populations of European ancestry, and further work is required to investigate whether the conclusions translate across ethnically diverse populations.

## Conclusions

This work identified genetic variants to instrument the effects of altered NPR2 and NPR3 signalling and function and employed them in the MR paradigm to generate genetic evidence supporting the protective effects of reduced NPR3 function on risk of CVD that are likely only partly mediated through blood pressure lowering. Triangulation with other evidence forms that make distinct assumptions is now required before channelling these findings towards clinical development efforts that would allow CNP to be harnessed as a therapeutic candidate for the treatment and prevention of CVD.

## Supplementary Information

**Additional file 1: Table S1.** Instrument SNPs used for NPR2- and NPR3-predicted height. **Table S2.** Mendelian randomization results of genome-wide, NPR2-, and NPR3-predicted height on cardiovascular disease risk, risk factors and comorbidities. **Table S3.** Instrument SNPs used for NPR3-predicted SBP. **Table S4.** Mendelian randomization results of genome-wide, and NPR3-predicted SBP on cardiovascular disease risk. **Table S5.** Colocalization results for the genetic associations between NPR2- and NPR3-predicted height and cardiovascular disease risk and risk factors. **Table S6.** Mendelian randomization statistical sensitivity analyses for the associations between NPR3-predicted height and cardiovascular disease risk and systolic blood pressure.

**Additional file 2: Figure S1.** Mendelian randomization estimates of genome-wide, NPR3-, and NPR2-predicted height on T2DM, CKD, and CVD risk. **Figure S2.** Mendelian randomization estimates of genome-wide, NPR3-, and NPR2-predicted height on blood pressure and blood lipid traits. **Figure S3, S4.** Regional plots of the genetic associations of height with CVD risk factors within +/-100kb of the NPR2 gene. [S3: HDL-C, S4: TG]. **Figure S5-S8.** Regional plots of the genetic associations of height with CVD risk and risk factors within +/-100kb of the NPR3 gene [S5: DBP, S6: Stroke, S7: CAD, S8: TG]. **Figure S9-S10.** Results of iteratively sampling 4 of the 12 NPR3-predicted height and 9695 height variants from throughout the genome and iteratively repeating random-effects inverse-variance weighted Mendelian randomization analysis 100 times. **Figure S11-13.** Comparative effects of each of the 12 NPR3 instrumental variables on height and cardiovascular outcomes[S11: CAD, S12: Heart failure, S13. Stroke]. **Figure S14.** Comparative effects of each of the 12 NPR3 instrumental variables on height and stroke.

## Data Availability

Only publicly available GWAS summary data were used in this work. Relevant publications and dataset access points are detailed in the reference list. The code used for this work may be obtained on request of the corresponding author.

## Abbreviations

CAD: Coronary artery disease
CI: Confidence interval
CKD: Chronic kidney disease
CNP: C-type natriuretic peptide
CVD: Cardiovascular disease
DBP: Diastolic blood pressure
HDL-C: High-density lipoprotein cholesterol
LD: Linkage disequilibrium
LDL-C: Low-density lipoprotein cholesterol
MR: Mendelian randomization
NPR2: Natriuretic peptide receptor 2
NPR3: Natriuretic peptide receptor 3
SBP: Systolic blood pressure
T2DM: Type-2 diabetes mellitus
TG: Triglyceriders
