# Supplementary material for: Genetic evidence implicating natriuretic peptide receptor-3 in cardiovascular disease risk: a Mendelian randomization study
Source: BMC Med. 2023 Apr 26;21:158. doi: 10.1186/s12916-023-02867-x (PMC10134514; doi:10.1186/s12916-023-02867-x)
Supplement: Supplementary file 1 — Additional file 1: Table S1. Instrument SNPs used for NPR2- and NPR3-predicted height. Table S2. Mendelian randomization results of genome-wide, NPR2-, and NPR3-predicted height on cardiovascular disease risk, risk factors and comorbidities. Table S3. Instrument SNPs used for NPR3-predicted SBP. Table S4. Mendelian randomization results of genome-wide, and NPR3-predicted SBP on cardiovascular disease risk. Table S5. Colocalization results for the genetic associations between NPR2- and NPR3-predicted height and cardiovascular disease risk and risk factors. Table S6. Mendelian randomization statistical sensitivity analyses for the associations between NPR3-predicted height and cardiovascular disease risk and systolic blood pressure. [file 12916_2023_2867_MOESM1_ESM.pdf]

SUPPLEMENTAL TABLES

Table S1. Instrument SNPs used for NPR2- and NPR3-predicted height

SNP	Location	EA	EAF	GX	GX SE	P	N	R^2	F
**Natriuretic peptide receptor 2 (NPR2)**									
rs867194	9:35705086	G	0.25	0.010	0.001	3E-21	2,083,833	0.00004	83
rs7847621	9:35780220	G	0.63	0.016	0.001	4E-57	2,018,522	0.00011	231
rs4879930	9:35825413	T	0.68	-0.009	0.001	1E-16	2,083,871	0.00003	67
rs12000024	9:35892051	G	0.22	0.010	0.001	2E-19	2,082,607	0.00003	73
**Natriuretic peptide receptor 3 (NPR3)**									
rs1471154	5:32600297	C	0.84	0.010	0.001	3E-13	2,066,029	0.00003	54
rs16890002	5:32638711	C	0.07	-0.013	0.002	1E-10	1,958,567	0.00002	42
rs17540044	5:32692238	C	0.23	0.028	0.001	2E-129	1,859,407	0.00028	523
rs976576	5:32722499	C	0.68	-0.007	0.001	2E-10	2,084,661	0.00002	40
rs696831	5:32725531	A	0.27	-0.008	0.001	3E-08	1,559,316	0.00002	35
rs1060559	5:32735408	T	0.10	0.017	0.002	5E-26	2,080,277	0.00005	101
rs3792752	5:32768634	G	0.27	0.031	0.001	3E-185	2,084,657	0.00037	766
rs1173771	5:32815028	G	0.61	-0.034	0.001	3E-280	2,084,650	0.00056	1160
rs9292469	5:32840210	T	0.35	-0.008	0.001	1E-15	2,083,376	0.00003	59
rs7722828	5:32843434	T	0.07	0.023	0.002	6E-37	2,083,729	0.00007	147
rs1472261	5:32887610	A	0.14	-0.009	0.001	8E-10	2,084,661	0.00002	36
rs2331101	5:32889284	T	0.27	-0.015	0.001	8E-48	2,081,059	0.00009	194

EA(F): effect allele (frequency), F statistic: a product of the size and precision of the genetic association $[R^2 x(N–2)/(1–R^2)]$, GX: standard deviation unit difference in height (cm) per effect allele; GX SE: standard error of GX, R^2: the proportion of the variance in height explained by the SNP $[2×EAF×(1–EAF)×GX^2]$, SNP: single-nucleotide polymorphism. Estimates ascertained from Yengo *et al.* (2022).

Table S2. Mendelian randomization results of genome-wide, NPR2-, and NPR3-predicted height on cardiovascular disease risk, risk factors and comorbidities

Outcome	Estimate unit	Exposure	Effect estimate (95%CI)	P
**CAD**	OR per SD	Height	0.882 (0.868; 0.895)	7.2E-65
		NPR2	0.768 (0.334; 1.768)	5.4E-01
		NPR3	0.745 (0.601; 0.924)	7.0E-03
**HF**	OR per SD	Height	1.03 (1.012; 1.048)	1.0E-03
		NPR2	1.178 (0.228; 6.086)	8.5E-01
		NPR3	0.772 (0.584; 1.02)	6.9E-02
**Stroke**	OR per SD	Height	0.969 (0.956; 0.981)	6.3E-06
		NPR2	0.829 (0.467; 1.471)	5.2E-01
		NPR3	0.692 (0.503; 0.953)	2.4E-02
**CVD**	OR per SD	Height	0.958 (0.877; 1.047)	3.5E-01
		NPR2	0.832 (0.528; 1.311)	4.3E-01
		NPR3	0.741 (0.637; 0.861)	1.0E-04
**T2DM**	OR per SD	Height	0.96 (0.944; 0.977)	6.8E-06
		NPR2	0.603 (0.216; 1.685)	3.4E-01
		NPR3	0.857 (0.718; 1.024)	9.0E-02
**CKD**	OR per SD	Height	1.073 (1.052; 1.093)	9.3E-12
		NPR2	1.043 (0.501; 2.175)	9.1E-01
		NPR3	0.893 (0.671; 1.189)	4.4E-01
**SBP**	Change per SD	Height	–0.04 (–0.047; –0.033)	6.6E-22
		NPR2	–0.026 (–0.326; 0.275)	8.7E-01
		NPR3	–0.546 (–0.886; –0.206)	2.0E-03
**DBP**	Change per SD	Height	–0.02 (–0.027; –0.012)	8.4E-07
		NPR2	–0.141 (–0.709; 0.427)	6.3E-01
		NPR3	–0.442 (–0.752; –0.131)	5.0E-03
**LDL-C**	Change per SD	Height	–0.062 (–0.07; –0.054)	5.9E-49
		NPR2	0.026 (–0.183; 0.235)	8.1E-01
		NPR3	0.025 (–0.019; 0.069)	2.7E-01
**HDL-C**	Change per SD	Height	–0.008 (–0.016; 0.000)	4.3E-02
		NPR2	–0.273 (–0.404; –0.142)	5.4E-05
		NPR3	–0.034 (–0.088; 0.020)	2.1E-01
**TG**	Change per SD	Height	–0.044 (–0.052; –0.037)	5.2E-26

	NPR2	0.245 (0.122; 0.367)	8.9E-05
	NPR3	0.060 (0.010; 0.110)	1.8E-02

CAD: coronary artery disease, CI: confidence interval, CKD: chronic kidney disease, CVD: cardiovascular disease (reflects a pooled CAD, heart failure and stroke effect estimate), DBP: diastolic blood pressure, HDL-C: high-density lipoprotein cholesterol, HF: heart failure, LDL-C: low-density lipoprotein cholesterol, NPR2: natriuretic peptide receptor 2, NPR3: natriuretic peptide receptor 3, OR: odds ratio, SBP: systolic blood pressure, SD: standard deviation, T2DM: type-2 diabetes mellitus, TG: triglycerides.

Table S3. Instrument SNPs used for NPR3-predicted systolic blood pressure

**SNP**	**Location**	**EA**	**EAF**	**GX**	**GX SE**	**P**	**N**	**R^2**	**F**
				**Natriuretic peptide receptor 3 (NPR3)**					
rs17540044	5:32692238	G	0.74	-0.018	0.003	8E-13	475,633	0.00013	62
rs1847018	5:32714661	C	0.88	-0.023	0.003	1E-11	475,633	0.00012	55
rs7733331	5:32828846	T	0.42	0.033	0.002	1E-47	475,633	0.00053	253
rs9292470	5:32872980	G	0.73	0.017	0.003	1E-11	475,633	0.00012	55

EA(F): effect allele (frequency), F statistic: a product of the size and precision of the genetic association [R^2x(N–2)/(1–R^2)], GX: standard deviation unit difference in systolic blood pressure (standard deviation units) per effect allele; GX SE: standard error of GX, R^2: the proportion of the variance in systolic blood pressure explained by the SNP [$2\times EAF\times(1–EAF)\times GX^2$], SNP: single-nucleotide polymorphism. Estimates ascertained from the Neale Lab (http://www.nealelab.is/uk-biobank/).

Table S4. Mendelian randomization results of genome-wide and NPR3-predicted SBP on cardiovascular disease risk

**Outcome**	**Exposure**	**Odds ratio (95% CI)** per SD change in SBP	**P**
**CAD**	SBP	0.562 (0.513; 0.616)	8.9E-32
	NPR3	0.681 (0.555; 0.836)	2.8E-04
**Heart failure**	SBP	0.705 (0.657; 0.757)	1.0E-20
	NPR3	0.816 (0.500; 1.331)	4.2E-01
**Stroke**	SBP	0.651 (0.608; 0.696)	1.5E-33
	NPR3	0.452 (0.347; 0.587)	6.2E-09
**CVD**	SBP	0.641 (0.561; 0.725)	3.5E-11
	NPR3	0.511 (0.439; 0.852)	3.7E-03

CAD: coronary artery disease, CI: confidence interval, CVD: cardiovascular disease (reflects a pooled CAD, heart failure and stroke effect estimate), NPR3: natriuretic peptide receptor 3, SBP: systolic blood pressure.

Table S5. Colocalization results for the genetic associations between NPR2- and NPR3-predicted height and cardiovascular disease risk and risk factors

Exposure	Outcome	SNPs	Posterior probability of causal variant hypotheses (PPH)					Lead SNP
			**0**: None	**1**: Exposure	**2**: Outcome	**3**: Distinct	**4**: Both	
NPR2	HDL-C	98	<0.01	0.04	<0.01	0.10	0.86	rs7847621
NPR2	TG	98	<0.01	0.25	<0.01	<0.01	0.75	rs7847621
NPR3	SBP	152	<0.01	<0.01	<0.01	0.01	0.99	rs1173771
NPR3	DBP	152	<0.01	<0.01	<0.01	<0.01	>0.99	rs1173771
NPR3	TG	153	<0.01	>0.99	<0.01	<0.01	<0.01	rs1173771
NPR3	CAD	153	<0.01	0.50	<0.01	0.01	0.49	rs1173771
NPR3	Stroke	153	<0.01	<0.01	<0.01	<0.01	0.99	rs1173771

Posterior probabilities of the following hypotheses are tested: **0**: no variants are causal; **1**: causal variant for exposure only; **2**: causal variant for outcome only; **3**: distinct causal variants for exposure and outcome (i.e., MR results may be due to LD between separate causal variants for the traits); **4**: shared causal variant for exposure and outcome (PPH4 >0.5 reflects colocalization in support of the MR evidence). CAD: coronary artery disease, DBP: diastolic blood pressure, HDL-C: high-density lipoprotein cholesterol, SBP: systolic blood pressure, SNP: single-nucleotide polymorphism, TG: triglycerides

Table S6. Mendelian randomization statistical sensitivity analyses for the associations between NPR3-predicted height and cardiovascular disease risk and systolic blood pressure.

**Outcome**	**Unit**	**Weighted Median**		**MR-Egger**			**I^2 (%)**
		GY (95% CI)	P	GY (95% CI)	P	P (intercept)	
**CAD**	OR	0.75 (0.6; 0.94)	1.1E-02	0.74 (0.48; 1.14)	1.7E-01	9.7E-01	46.5 (0.00; 72.6)
**Heart failure**	OR	0.75 (0.53; 1.05)	9.7E-02	0.78 (0.45; 1.37)	3.9E-01	9.6E-01	0.00 (0.00; 58.3)
**Stroke**	OR	0.51 (0.36; 0.72)	1.8E-04	0.62 (0.33; 1.18)	1.5E-01	7.0E-01	64.0 (33.2; 80.6)
**SBP**	SD	-0.71 (-0.87; -0.55)	1.1E-17	-0.71 (-1.36; -0.06)	3.3E-02	6.0E-01	93.9 (91.0; 95.8)

Abbreviations: CAD: coronary artery disease, CI: confidence interval, OR: odds ratio, SBP: systolic blood pressure, SD: standard deviation. All estimates are given per 1-SD increase in NPR3-predicted height.
