# Supplementary material for: Genetic evidence implicating natriuretic peptide receptor-3 in cardiovascular disease risk: a Mendelian randomization study
Source: BMC Med. 2023 Apr 26;21:158. doi: 10.1186/s12916-023-02867-x (PMC10134514; doi:10.1186/s12916-023-02867-x)
Supplement: Supplementary file 2 — Additional file 2: Figure S1. Mendelian randomization estimates of genome-wide, NPR3-, and NPR2-predicted height on T2DM, CKD, and CVD risk. Figure S2. Mendelian randomization estimates of genome-wide, NPR3-, and NPR2-predicted height on blood pressure and blood lipid traits. Figure S3, S4. Regional plots of the genetic associations of height with CVD risk factors within +/-100kb of the NPR2 gene. [S3: HDL-C, S4: TG]. Figure S5-S8. Regional plots of the genetic associations of height with CVD risk and risk factors within +/-100kb of the NPR3 gene [S5: DBP, S6: Stroke, S7: CAD, S8: TG]. Figure S9-S10. Results of iteratively sampling 4 of the 12 NPR3-predicted height and 9695 height variants from throughout the genome and iteratively repeating random-effects inverse-variance weighted Mendelian randomization analysis 100 times. Figure S11-13. Comparative effects of each of the 12 NPR3 instrumental variables on height and cardiovascular outcomes[S11: CAD, S12: Heart failure, S13. Stroke]. Figure S14. Comparative effects of each of the 12 NPR3 instrumental variables on height and stroke. [file 12916_2023_2867_MOESM2_ESM.pdf]

SUPPLEMENTAL FIGURES

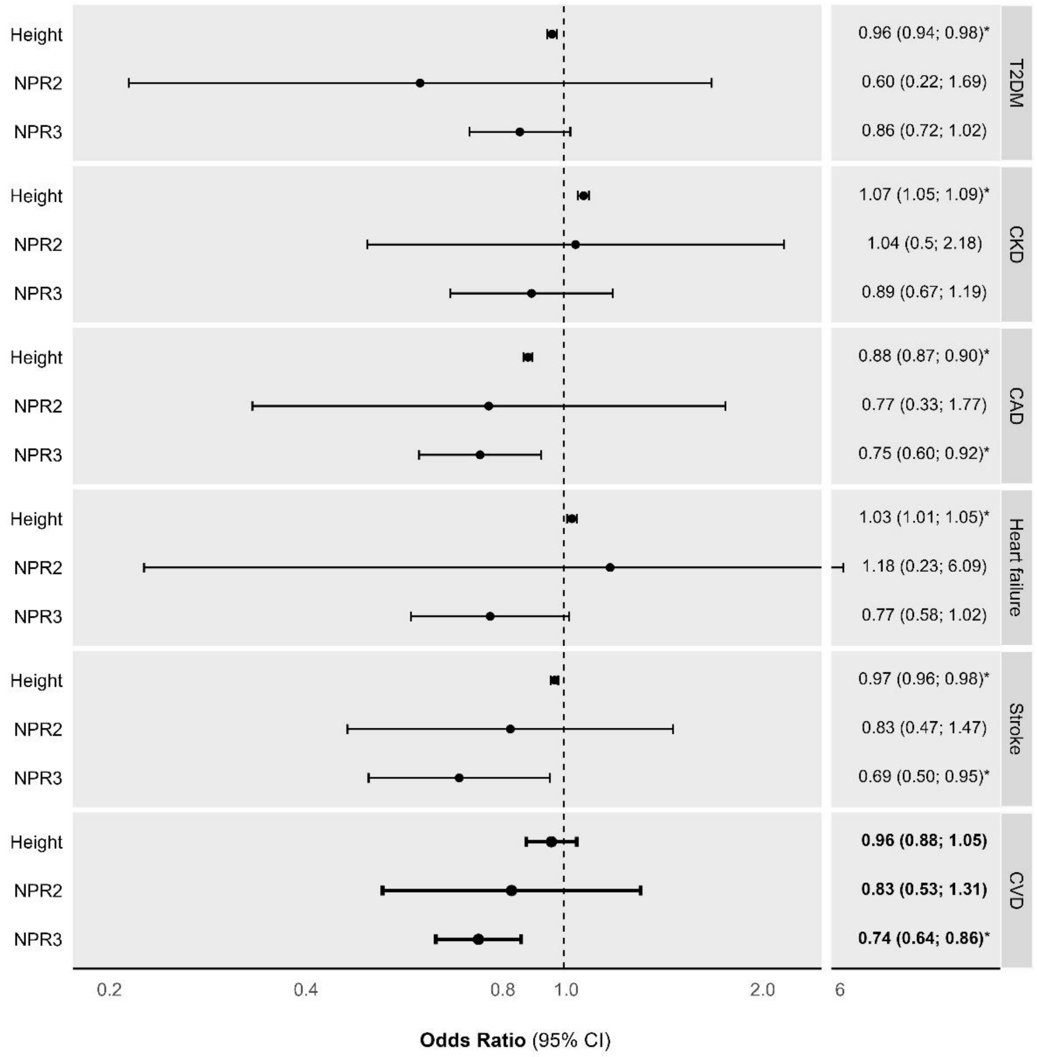

Figure S1. Mendelian randomization estimates of genome-wide, NPR3-, and NPR2-predicted height on T2DM, CKD, and CVD risk.

Effect estimates are scaled per standard deviation increased height (~9.2cm). * denotes p<.05. CAD: coronary artery disease, CI: confidence interval, CKD: chronic kidney disease, CVD: cardiovascular disease (reflects a pooled CAD, heart failure and stroke estimate), NPR2: natriuretic peptide receptor 2, NPR3: natriuretic peptide receptor 3, T2DM: type-2 diabetes mellitus.

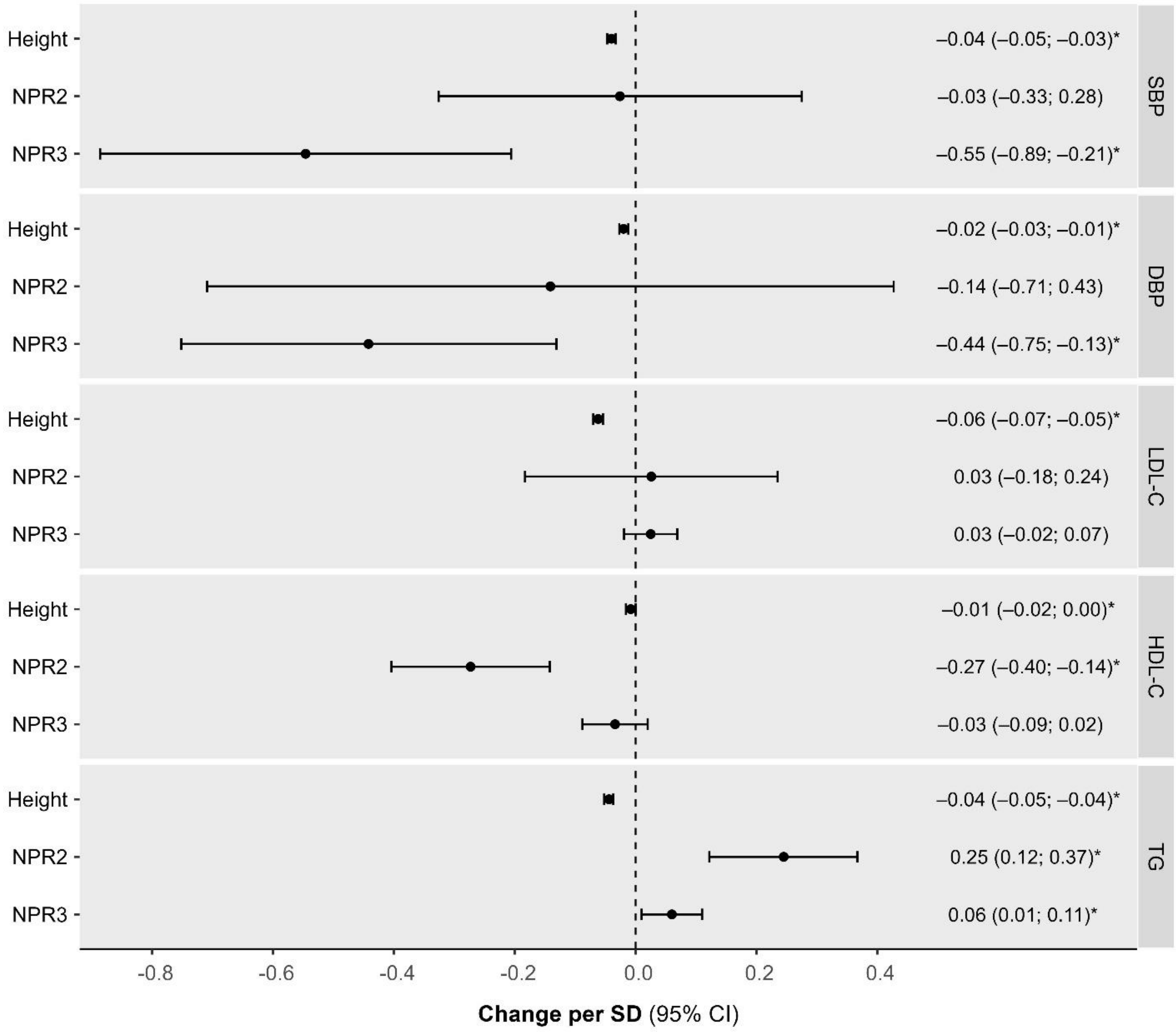

Figure S2. Mendelian randomization estimates of genome-wide, NPR3, and NPR2 predicted height on blood pressure and blood lipid traits.

Effect estimates are interpreted as standard deviation (SD) unit difference per 1-SD higher height (~9.2cm). * denotes p<.05. CI: confidence interval, DBP: diastolic blood pressure, HDL-C: high-density lipoprotein cholesterol, LDL-C: low-density lipoprotein cholesterol, NPR2: natriuretic peptide receptor 2, NPR3: natriuretic peptide receptor 3, SBP: systolic blood pressure, SD: standard deviation, TG: triglycerides.

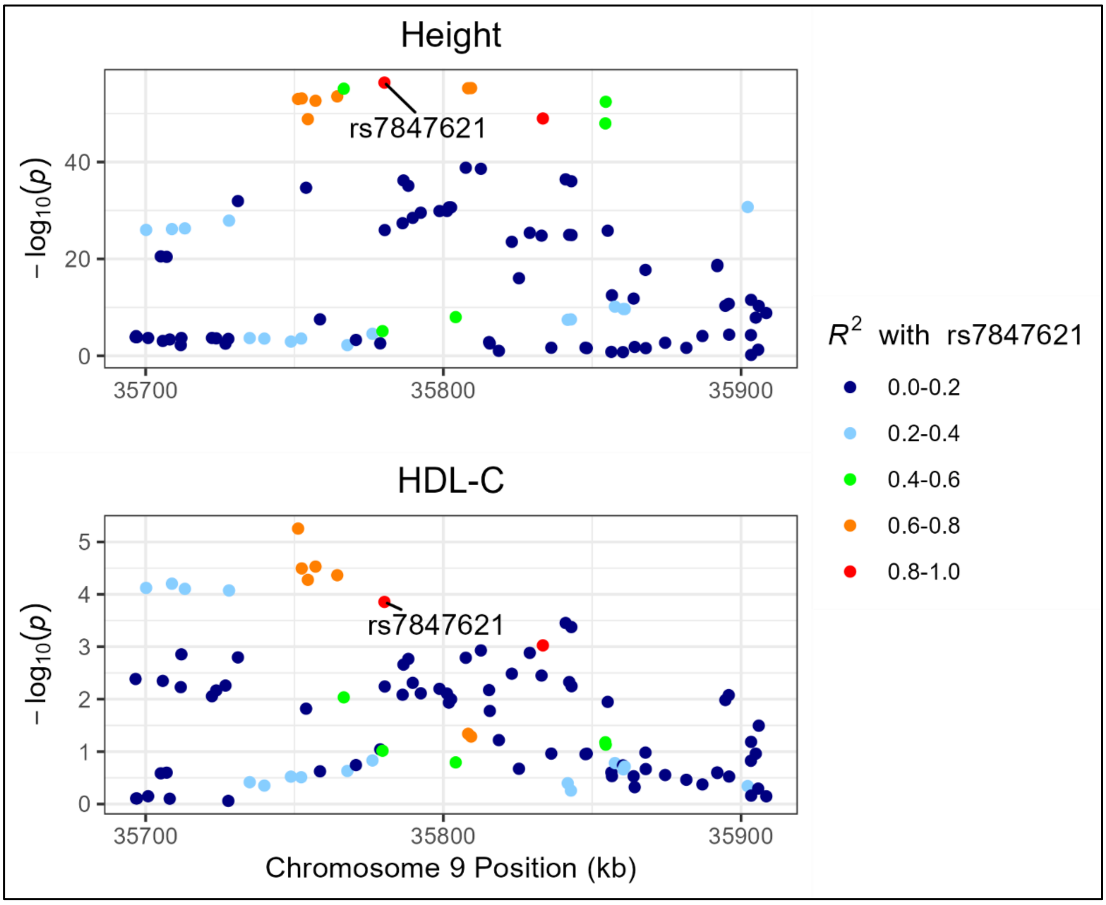

Fig. S3. Regional plot of the genetic associations of height (upper panel) and high-density lipoprotein cholesterol (HDL-C; lower panel) within +/-100kb of the *NPR2* gene.

The x-axis shows genomic position (build hg19) for each variant, and y-axis shows the –log10(p-value) for the association. Colour denotes the linkage disequilibrium R^2 with rs7847621, the most likely shared causal variant based on colocalization results.

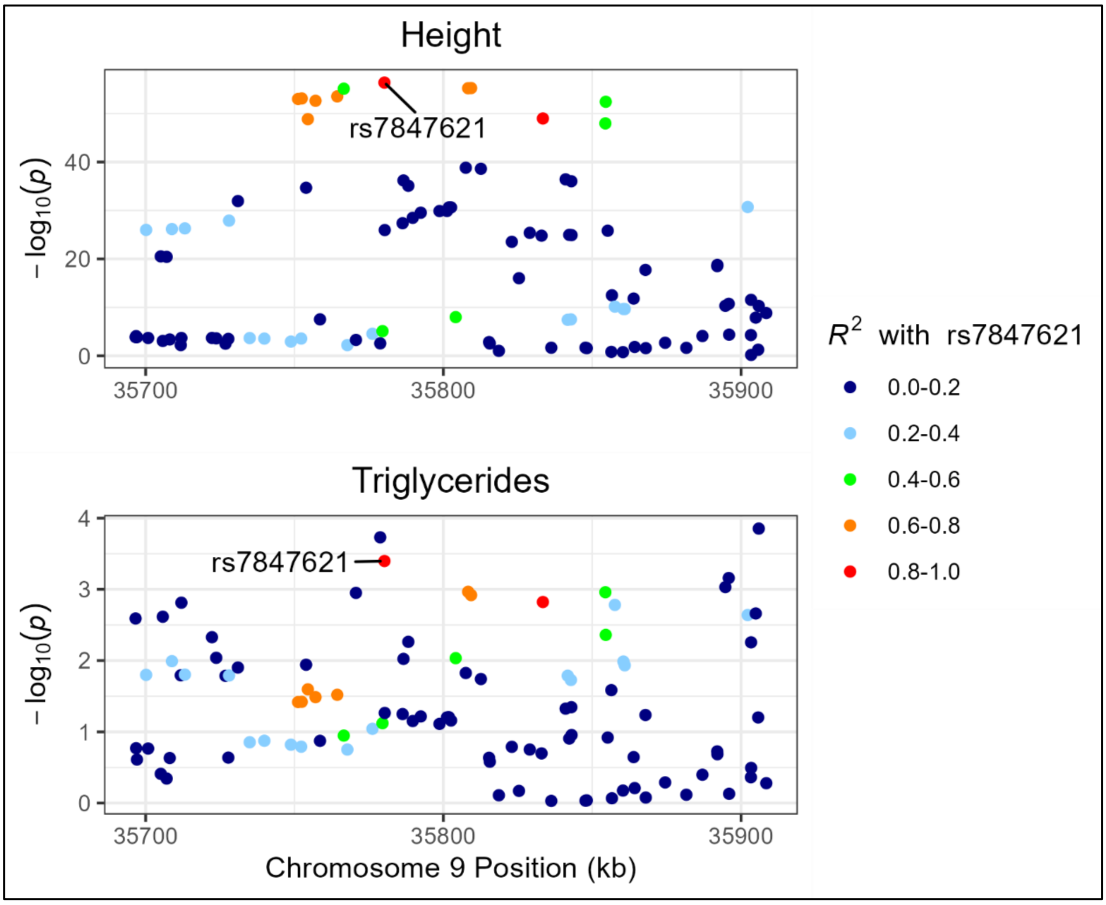

Fig. S4. Regional plot of the genetic associations of height (upper panel) and triglycerides (lower panel) within +/-100kb of the *NPR2* gene.

The x-axis shows genomic position (build hg19) for each variant, and y-axis shows the –log10(p-value) for the association. Colour denotes the linkage disequilibrium R^2 with rs7847621, the most likely shared causal variant based on colocalization results.

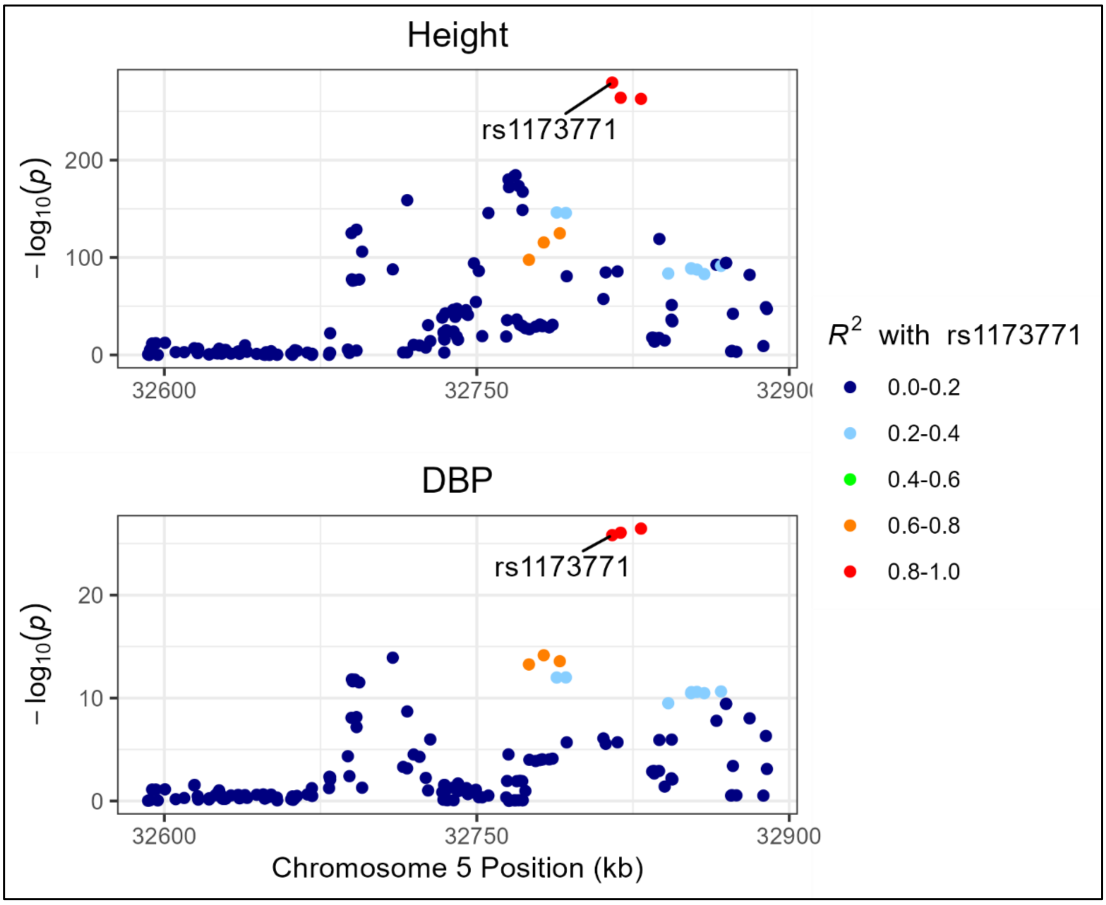

Fig. S5. Regional plot of the genetic associations of height (upper panel) and diastolic blood pressure (DBP; lower panel) within +/-100kb of the *NPR3* gene.

The x-axis shows genomic position (build hg19) for each variant, and y-axis shows the –log10(p-value) for the association. Colour denotes the linkage disequilibrium R^2 with rs1173771, the most likely shared causal variant based on colocalization results.

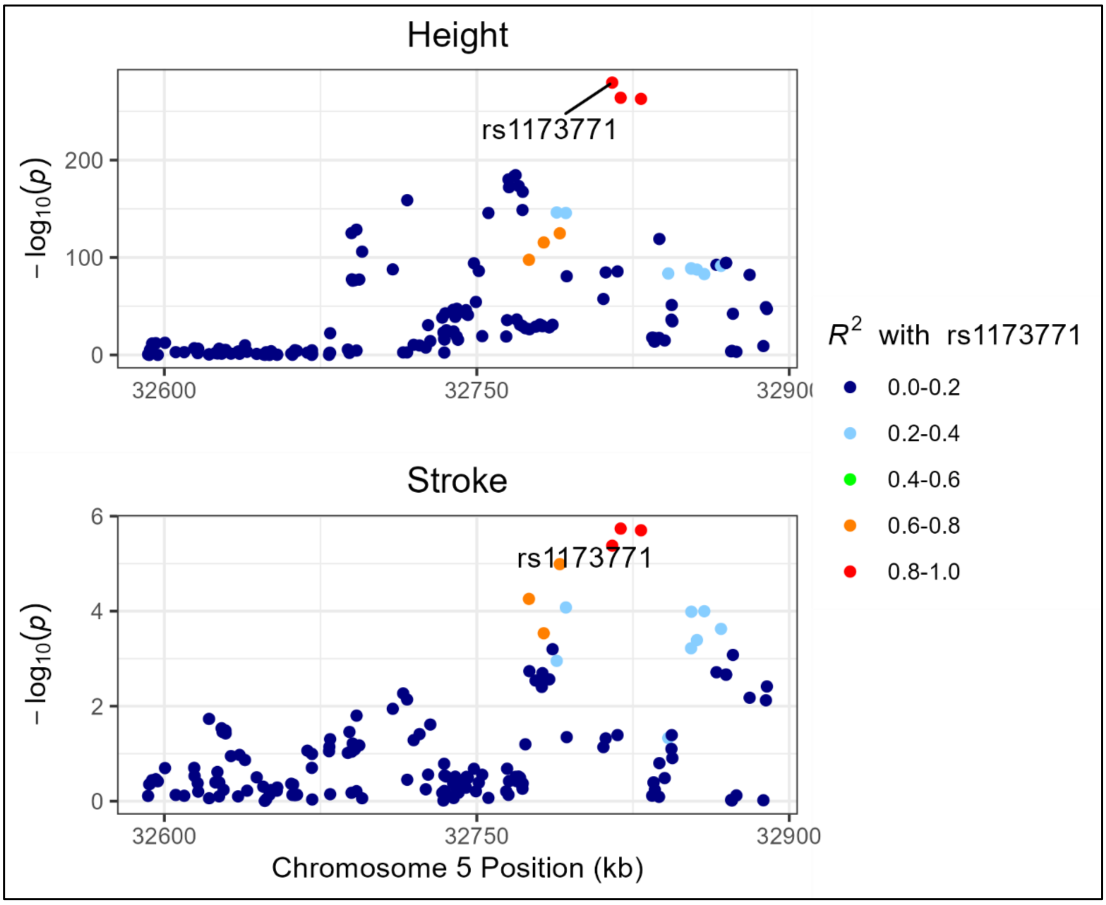

Fig. S6. Regional plot of the genetic associations of height (upper panel) and stroke (lower panel) within +/-100kb of the *NPR3* gene.

The x-axis shows genomic position (build hg19) for each variant, and y-axis shows the –log10(p-value) for the association. Colour denotes the linkage disequilibrium R^2 with rs1173771, the most likely shared causal variant based on colocalization results.

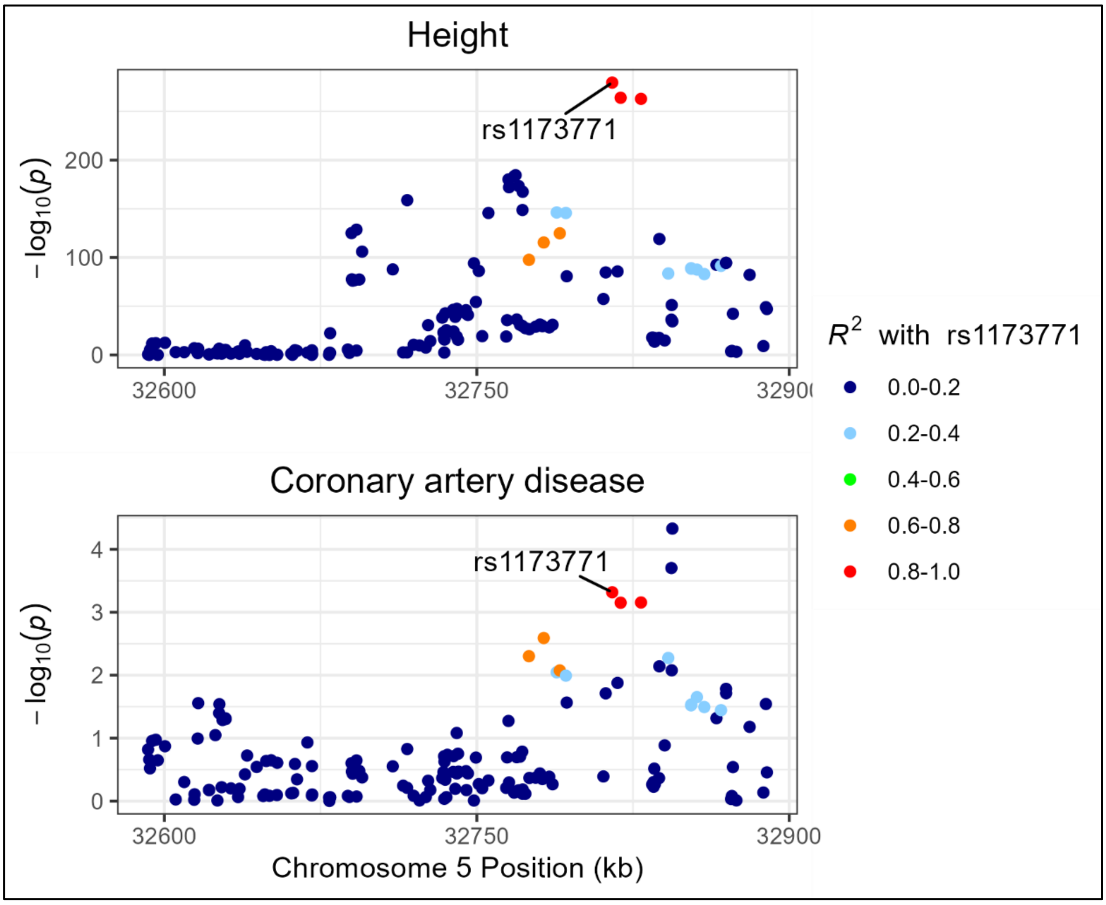

Fig. S7. Regional plot of the genetic associations of height (upper panel) and coronary artery disease (lower panel) within +/-100kb of the *NPR3* gene.

The x-axis shows genomic position (build hg19) for each variant, and y-axis shows the –log10(p-value) for the association. Colour denotes the linkage disequilibrium R^2 with rs1173771, the most likely shared causal variant based on colocalization results.

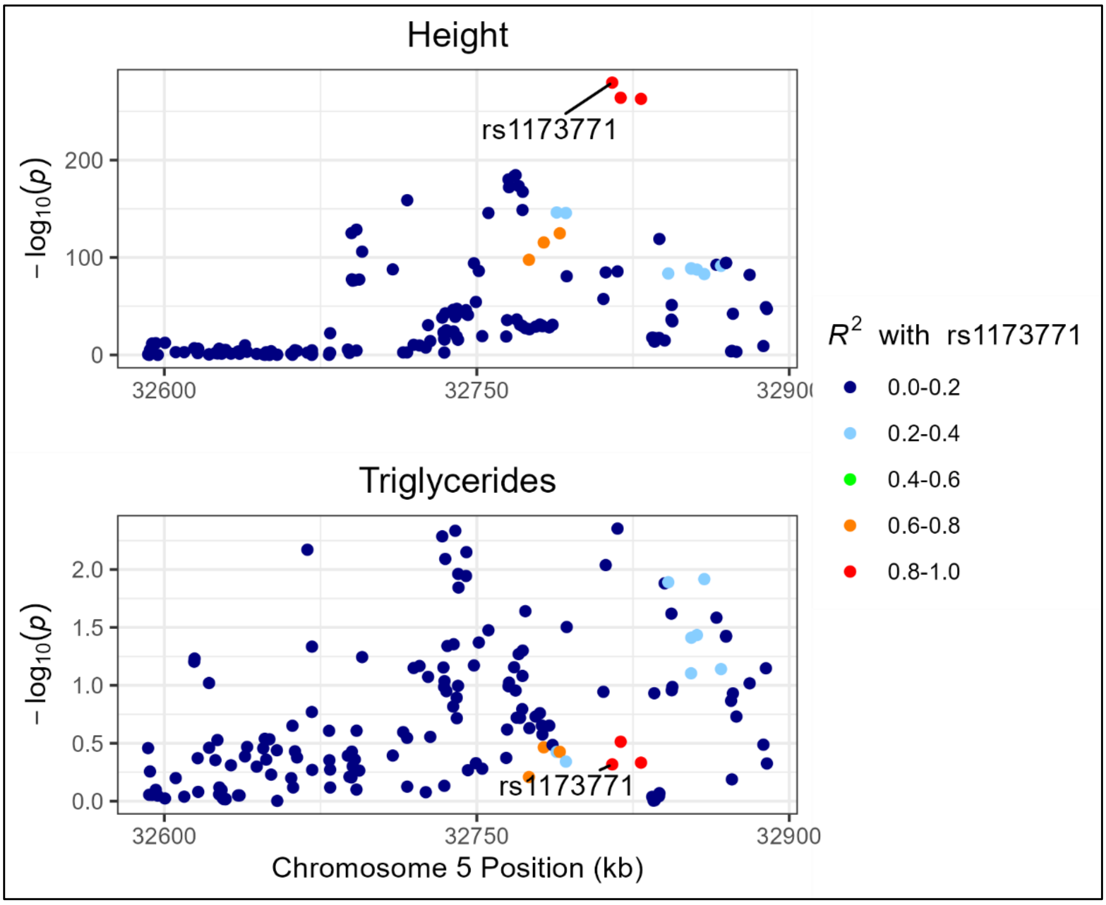

Fig. S8. Regional plot of the genetic associations of height (upper panel) and triglycerides (lower panel) within +/-100kb of the *NPR3* gene.

The x-axis shows genomic position (build hg19) for each variant, and y-axis shows the –log10(p-value) for the association. Colour denotes the linkage disequilibrium R^2 with rs1173771, the most likely shared causal variant based on colocalization results.

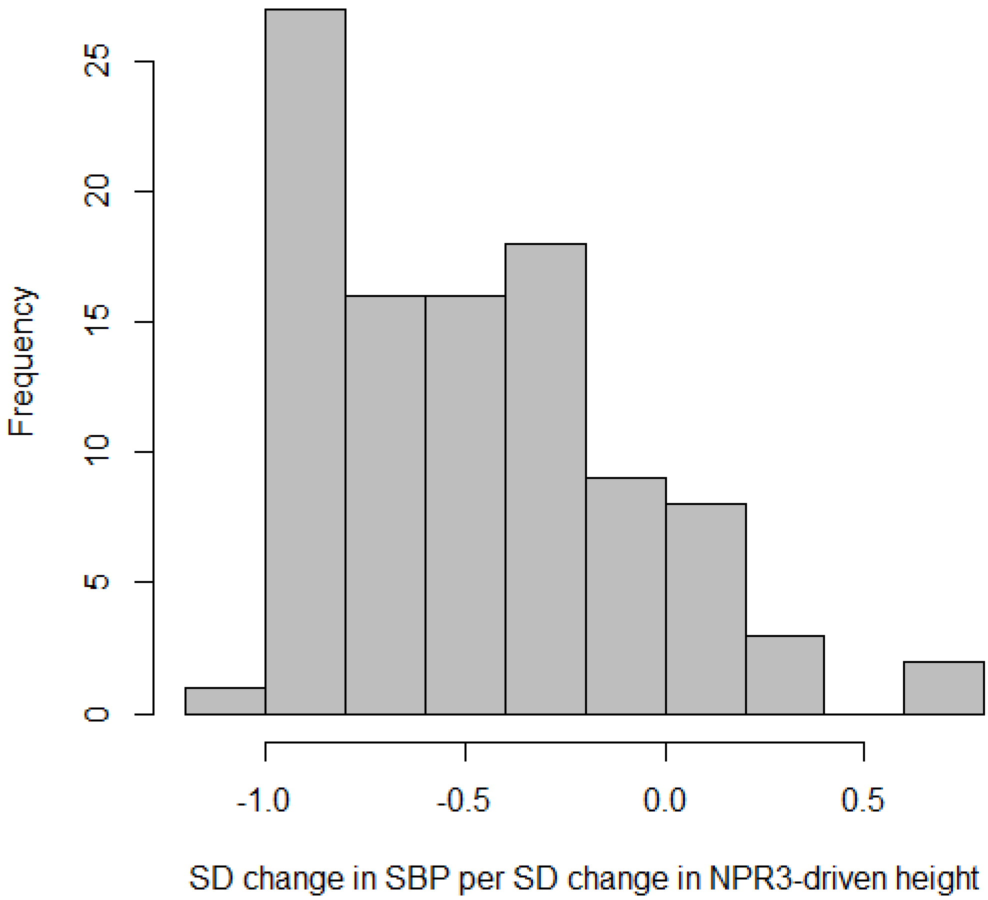

Fig. S9. Results of iteratively sampling 4 of the 12 NPR3 variants 100 times and repeating random-effects inverse-variance weighted Mendelian randomization analysis for the outcome of systolic blood pressure.

SBP: systolic blood pressure, SD: standard deviation.

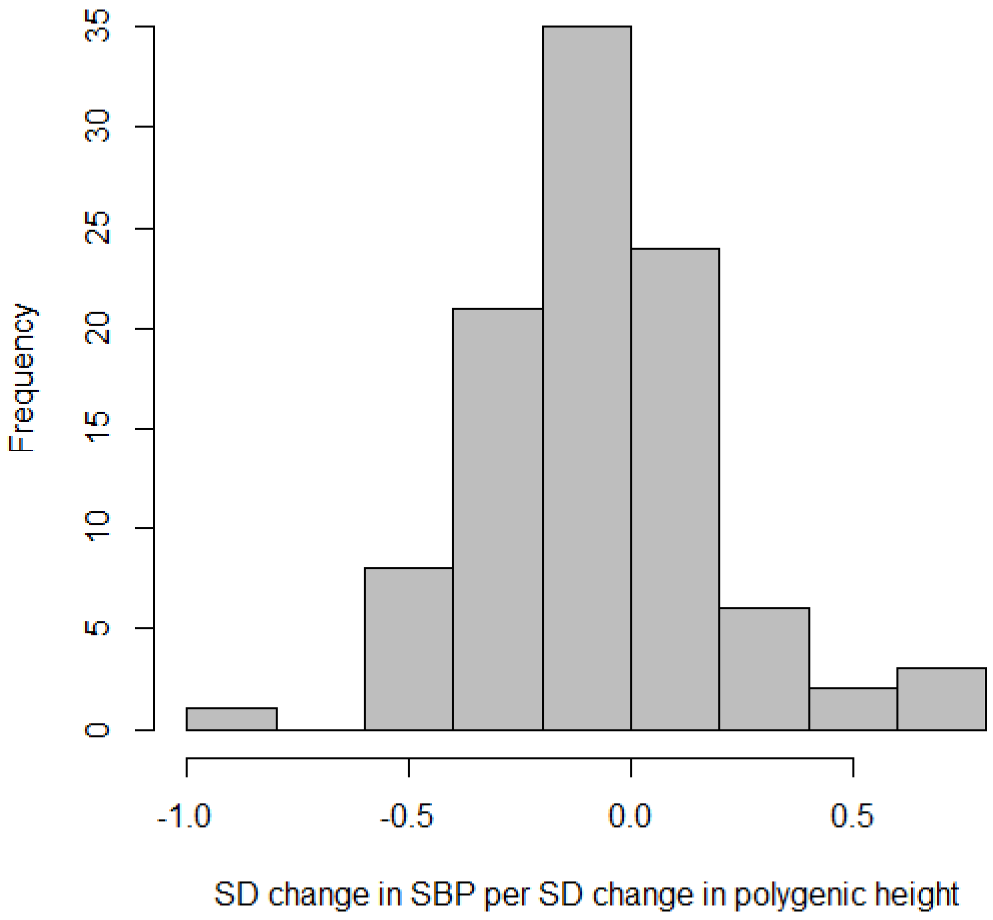

Fig. S10. Results of iteratively sampling 4 of the 9,695 height variants from across the genome 100 times and repeating random-effects inverse-variance weighted Mendelian randomization analysis for the outcome of systolic blood pressure.

SBP: systolic blood pressure, SD: standard deviation.

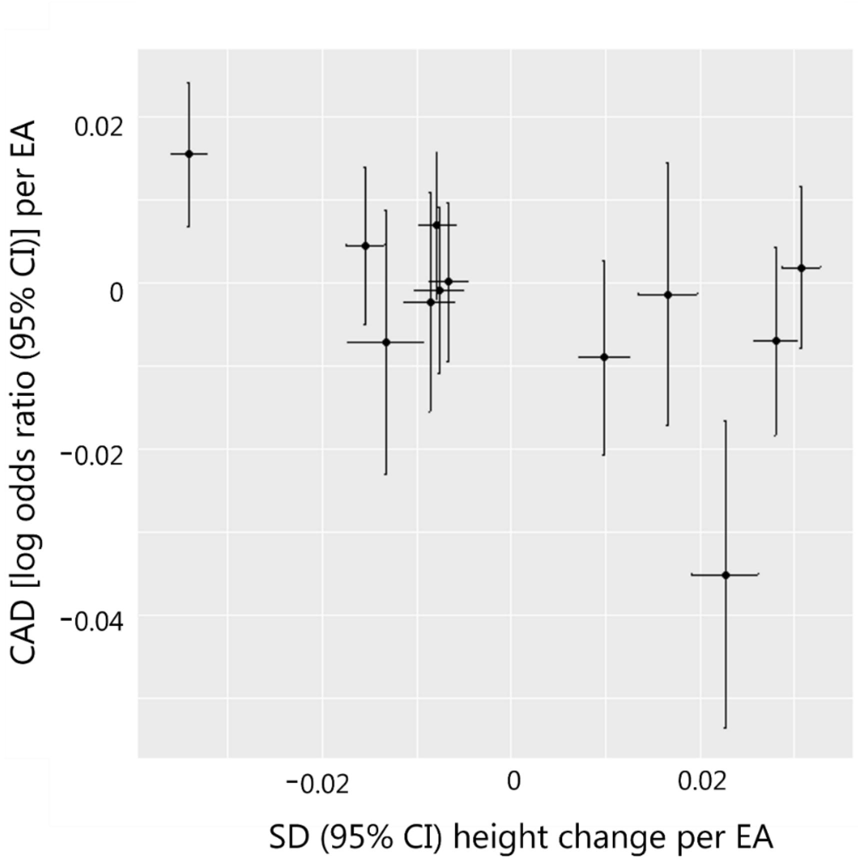

Fig. S11. Comparative effects of each of the 12 NPR3 instrumental variables on height (x-axis) and CAD (y-axis).

CAD: coronary artery disease, CI: confidence interval, EA: effect allele, NPR3: natriuretic peptide receptor 3, SD: standard deviation

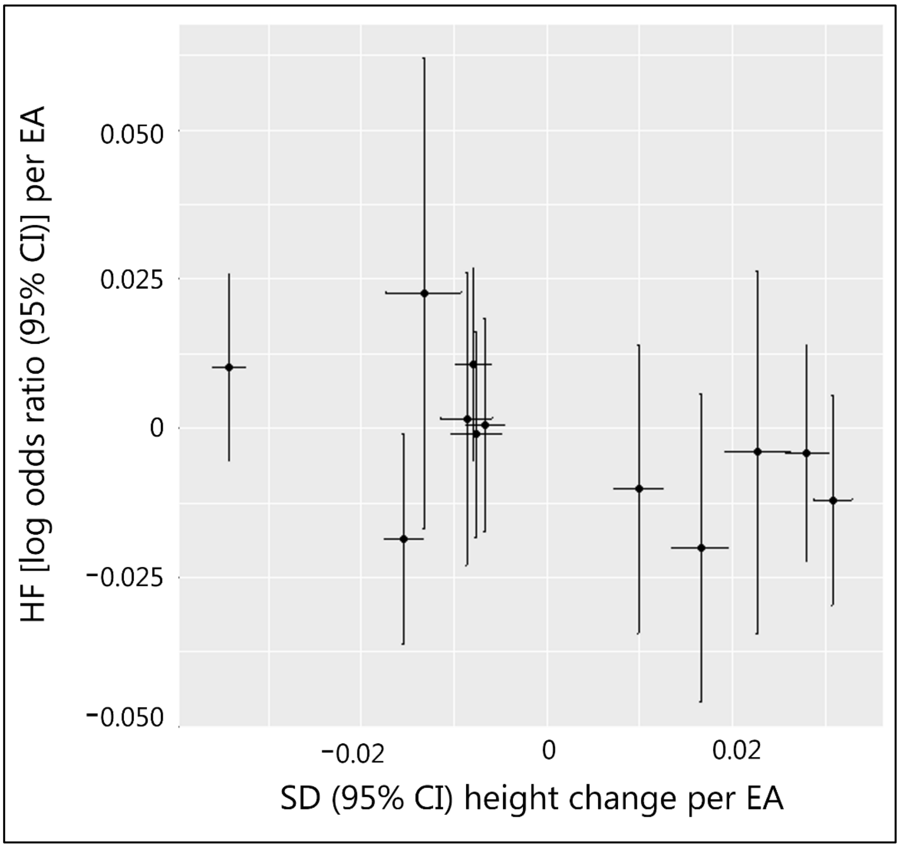

Fig. S12. Comparative effects of each of the 12 NPR3 instrumental variables on height (x-axis) and heart failure (y-axis).

CI: confidence interval, EA: effect allele, NPR3: natriuretic peptide receptor 3, SD: standard deviation

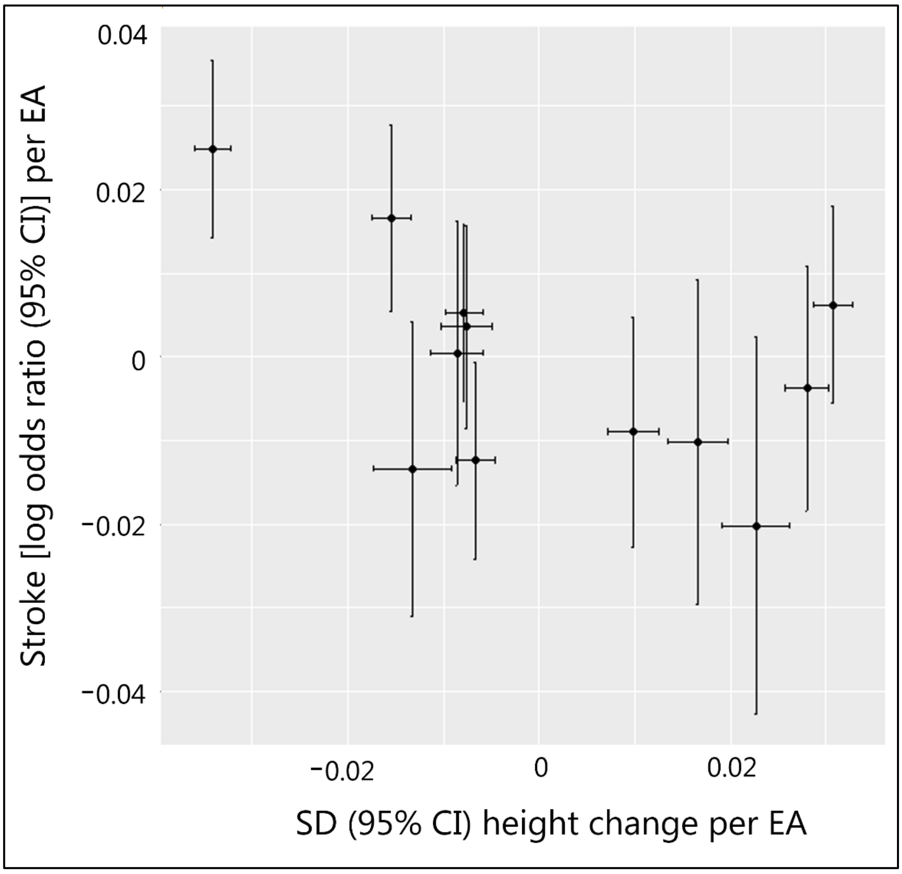

Fig. S13. Comparative effects of each of the 12 NPR3 instrumental variables on height (x-axis) and stroke (y-axis).

CI: confidence interval, EA: effect allele, NPR3: natriuretic peptide receptor 3, SD: standard deviation

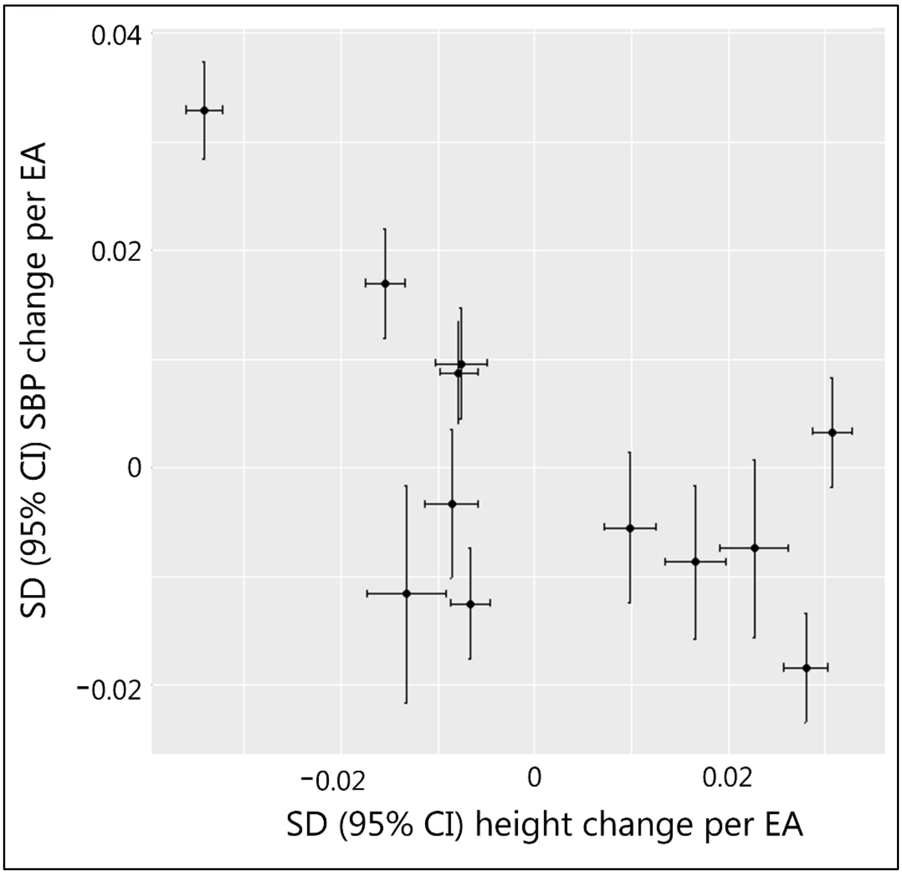

Fig. S14. Comparative effects of each of the 12 NPR3 instrumental variables on height (x-axis) and stroke (y-axis).

CI: confidence interval, EA: effect allele, NPR3: natriuretic peptide receptor 3, SBP: systolic blood pressure, SD: standard deviation
